# Substance P Immunoreactivity Exhibits Frequent Colocalization with Kisspeptin and Neurokinin B in the Human Infundibular Region

**DOI:** 10.1371/journal.pone.0072369

**Published:** 2013-08-19

**Authors:** Erik Hrabovszky, Beáta Á. Borsay, Kálmán Rácz, László Herczeg, Philippe Ciofi, Stephen R. Bloom, Mohammad A. Ghatei, Waljit S. Dhillo, Zsolt Liposits

**Affiliations:** 1 Laboratory of Endocrine Neurobiology, Institute of Experimental Medicine, Hungarian Academy of Sciences, Budapest, Hungary; 2 Department of Forensic Medicine, Faculty of Medicine of the University of Debrecen, Debrecen, Hungary; 3 INSERM U862, Neurocentre Magendie, Bordeaux, France; 4 Department of Investigative Medicine, Hammersmith Hospital, Imperial College London, London, United Kingdom; 5 Department of Neuroscience, Faculty of Information Technology, Pázmány Péter Catholic University, Budapest, Hungary; John Hopkins University School of Medicine, United States of America

## Abstract

Neurons synthesizing neurokinin B (NKB) and kisspeptin (KP) in the hypothalamic arcuate nucleus represent important upstream regulators of pulsatile gonadotropin-releasing hormone (GnRH) neurosecretion. In search of neuropeptides co-expressed in analogous neurons of the human infundibular nucleus (Inf), we have carried out immunohistochemical studies of the tachykinin peptide Substance P (SP) in autopsy samples from men (21-78 years) and postmenopausal (53-83 years) women. Significantly higher numbers of SP-immunoreactive (IR) neurons and darker labeling were observed in the Inf of postmenopausal women than in age-matched men. Triple-immunofluorescent studies localized SP immunoreactivity to considerable subsets of KP-IR and NKB-IR axons and perikarya in the infundibular region. In postmenopausal women, 25.1% of NKB-IR and 30.6% of KP-IR perikarya contained SP and 16.5% of all immunolabeled cell bodies were triple-labeled. Triple-, double- and single-labeled SP-IR axons innervated densely the portal capillaries of the infundibular stalk. In quadruple-labeled sections, these axons formed occasional contacts with GnRH-IR axons. Presence of SP in NKB and KP neurons increases the functional complexity of the putative pulse generator network. First, it is possible that SP modulates the effects of KP and NKB in axo-somatic and axo-dendritic afferents to GnRH neurons. Intrinsic SP may also affect the activity and/or neuropeptide release of NKB and KP neurons via autocrine/paracrine actions. In the infundibular stalk, SP may influence the KP and NKB secretory output via additional autocrine/paracrine mechanisms or regulate GnRH neurosecretion directly. Finally, possible co-release of SP with KP and NKB into the portal circulation could underlie further actions on adenohypophysial gonadotrophs.

## Introduction

The hypothalamic arcuate nucleus (ARC) plays a pivotal role in reproductive regulation [1]. A particularly important peptidergic cell group in the sheep [2], rodent [3] and goat [4] ARC co-synthesizes kisspeptin (KP), neurokinin B (NKB) and dynorphin; these cells, lately referred to as ‘KNDy neurons’ [5], have been implicated in sex steroid negative feedback to the reproductive axis [6] and, as pacemaker cells, in the pulsatile neurosecretion of gonadotropin-releasing hormone (GnRH) [3,4,7].

While it is tempting to make predictions about the neuroanatomy and functions of a putative human ‘KNDy’ neuronal system from the known properties of the rodent or ruminant KNDy neurons, direct extrapolation from the results of animal experiments is not always possible as there can be significant species differences in the hypothalamic regulation of reproduction. Notably, several neurochemical differences have already been revealed between the human and the animal KP and NKB neuronal systems. Accordingly, results of our recent immunohistochemical studies show that the cellular colocalization between KP and NKB in young human males (<50 years) is of much lower extent than reported previously for laboratory animals; only 33% of NKB neurons and 75% of KP neurons expressed the other neuropeptide at levels accessible for immunohistochemical detection [8]. Somewhat surprisingly and unlike reported in rodents [3] and ruminants [2,9], we were unable to detect dynorphin immunoreactivity in the cell bodies and in most fibers of KP-immunoreactive (IR) neurons [8], although earlier *in situ* hybridization studies demonstrated the presence of dynorphin expressing neurons in the Inf [10]. Differences between animal and human neuroanatomy and reproductive physiology call for further studies to identify other neurotransmitters/neuromodulators which may co-localize specifically with the human KP and NKB neuronal systems.

In the present study we have addressed a potential overlap between the NKB and/or KP cell populations and those expressing the preprotachykinin A gene product, Substance P (SP) in the human Inf, by using immunohistochemical experiments on *post mortem* tissue samples. SP is an eleven-amino acid-neuropeptide, with the highest affinity to the G protein-coupled NK1 receptor, whereas neurokinin A, another derivative of the same preprotachykinin A gene, acts via the NK2 receptor [11]. As reviewed recently [12], SP can influence reproduction via acting at the hypothalamic, pituitary and gonadal levels of the reproductive axis. Although more attention has been paid to NKB than to SP in the context of reproductive regulation [12], results of previous *in situ* hybridization experiments by Rance and Young provided evidence that NKB and SP neurons of the Inf show highly similar distribution patterns and postmenopausal hypertrophy, furthermore, exhibit similarly increased numbers and higher mRNA levels in postmenopausal *vs.* premenopausal women [13]. We have carried out the immunohistochemical colocalization experiments on tissue samples from postmenopausal females as well as from males of variable ages, in view that KP and NKB levels and the extent of their colocalization vary significantly with age [14] and sex [15], furthermore, the regulation of SP expression in this region is also highly sex steroid dependent [13].

## Materials and Methods

### Ethics statement

Human brain tissue samples were obtained at autopsy from the Forensic Medicine Department of the University of Debrecen, with the permission of the Regional Committee of Science and Research Ethics (DEOEC RKEB/IKEB: 3183-2010) and according to the Hungarian Law (1997 CLIV and 18/1998/XII.27. EU¨M Decree/). All personal data were anonymized.

### Human subjects

Hypothalamic tissue blocks from fourteen male (21-78 years) and eight postmenopausal female (53-83 years) subjects were used. Tissue collection, section preparation and sample processing for immunohistochemistry were carried out as in recent studies on the human hypothalamic KP and NKB systems [8,14-16]. Selection criteria included sudden causes of death, lack of history of neurological and endocrine disorders and *post mortem* intervals below 48h. Male samples were subdivided further to arbitrary ‘young’ (<50 ys; N=8) and ‘aged’ (≥50 ys; N=6) subgroups, to allow similar statistical comparisons as in earlier studies addressing the sexual dimorphism [16] and aging-related changes in men [14], of the KP and NKB systems.

### Tissue preparation for immunohistochemistry

Following dissection, the hypothalamic tissue blocks were rinsed briefly with running tap water and then, immersed into 4% formaldehyde in 0.1M phosphate buffer saline (PBS; pH 7.4) for 7-14 days at 4°C. Prior to section preparation, the fixed hypothalami were trimmed further to include the optic chiasm rostrally, the mammillary bodies caudally and the anterior commissure dorsally [15-17]. Sagittal cuts were made 2cm lateral from the midsagittal plane on both sides and then, the blocks were cut in halves and infiltrated with 20% sucrose for 5 days at 4°C. The right hemihypothalami were placed in a freezing mold, surrounded with Jung tissue freezing medium (Leica Microsystems, Nussloch Gmbh, Germany; diluted 1:1 with 0.9% sodium chloride solution), snap-frozen on powdered dry ice, and sectioned coronally at 30μm with a Leica SM 2000R freezing microtome (Leica Microsystems). The sections were stored permanently in anti-freeze solution (30% ethylene glycol; 25% glycerol; 0.05 M phosphate buffer; pH 7.4) at -20^°^C.

### Tissue pretreatments

Every 72^nd^ section of the Inf (2-3 Inf sections per subject) was processed in each immunohistochemical study. For pretreatment, they were rinsed in PBS and incubated with a mixture of 0.5% H_2_O_2_ and 0.2% Triton X-100 for 30 min. Then, antigen retrieval was carried out with 0.1M citrate buffer (pH=6.0) at 80°C for 30 min. In immunofluorescent experiments, the sections were additionally treated with Sudan black to reduce tissue autofluorescence caused by lipofuscin deposits [18].

### Peroxidase-based immunohistochemistry

Incubation of sections in the primary antisera for 48 h at 4°C was followed by biotinylated secondary antibodies (Jackson ImmunoResearch Laboratories, West Grove, PA, USA; 1:500) and the ABC Elite reagent (Vector, Burlingame, CA; 1:1000) for 60 min each. The peroxidase signal was visualized with nickel-intensified diaminobenzidine chromogen and then, post-intensified with silver-gold [19]. KP immunoreactivity was detected with a sheep polyclonal antiserum (GQ2; 1:150,000) against human kisspeptin-54. This antiserum recognizes human kisspeptin-54, kisspeptin-14 and kisspeptin-10 and shows virtually no cross-reactivity (<0.01%) with other related human RF-amide peptide, including prolactin releasing peptide, neuropeptide FF, neuropeptide AF and RF-amide related peptides (RFRP1, RFRP2, RFRP3) [20]. The GQ2 antibodies were used successfully in previous immunohistochemical experiments to study the distribution of KP neurons and their connectivity to GnRH cells in the rhesus monkey [21,22] and the human [8,14-16]. To detect NKB synthesizing neurons, a previously characterized rabbit polyclonal antiserum (IS-682; P. Ciofi; 1:100,000) against the C-terminal 28 amino acids of human pro-NKB was used [8,14-16,22]. SP immunoreactivity was visualized with a rat monoclonal antibody against SP conjugated to bovine serum albumin [23] (Serotec #8450-0505; Bio-Rad Laboratories, Inc., Hercules, CA; 1:30,000), or alternatively, with a rabbit polyclonal antiserum (#505D3) directed toward the carboxyl terminus of SP (kind gift from Dr. P. Petrusz, Department of Anatomy, University of North Carolina, Chapel Hill, NC; 1:150,000) which requires an amidated carboxyl terminus group for recognition and shows less than 0.05% cross-reactivity with either NKA or NKB [24].

### Simultaneous visualization of SP, KP and NKB with triple-immunofluorescent labeling

A series of sections was used to study the putative colocalization between SP, KP and NKB immunoreactivities. In order to maximize the sensitivity of SP and KP detection, tyramide signal amplification was used for both. First, SP was detected using the sequential incubation of sections in rat SP antibodies (1:10,000; 48h; 4^°^C) and anti-rat-peroxidase conjugates (Jackson ImmunoResearch Laboratories; 1:250; 120 min). Then, FITC-tyramide [25] (diluted 1:500 with 0.05M Tris-HCl buffer, pH 7.6, containing 0.003% H_2_O_2_; 30 min) was deposited on the peroxidase sites. Subsequently, the sections were treated for 30 min with 0.5% H_2_O_2_ in PBS, to inactivate peroxidase, and processed further for the detection of the other two neuropeptides, using the following incubation steps: a cocktail of sheep anti-KP (1:40,000) and rabbit anti-NKB (1:1000) primary antibodies (48h, 4C), biotinylated anti-sheep IgG (Jackson ImmunoResearch Laboratories; 1:500; 1h), ABC Elite reagent (Vector; 1:1000; 1h), biotin-tyramide (diluted 1:1000 with 0.05M Tris-HCl buffer, pH 7.6, containing 0.003% H_2_O_2_; 30 min), and finally, a cocktail of avidin-Cy3 (1:1000) and anti-rabbit-Cy5 (1:500) (ea. from Jackson ImmunoResearch Laboratories; 12h).

### Quadruple-labeling to study the relationship of SP-IR, KP-IR and NKB-IR fibers to GnRH-IR axons around the hypophysial portal vasculature

A series of sections from the infundibular stalk was used to address a putative axo-axonal communication between SP-IR, KP-IR and NKB-IR axons and hypophysiotropic GnRH projections. First, the triple-labeling procedure from above was carried out. Then, the sections were incubated overnight in a guinea pig GnRH antiserum (#1018; 1:3000) generated and characterized in our laboratory [16], followed by anti-guinea pig-AMCA conjugates (Jackson ImmunoResearch Laboratories; 1:50; 12h).

### Specificity controls

Various control approaches were used to confirm the specificity of immunohistochemical results. In case of KP and NKB, previously detailed specificity tests included the comparative analysis of the immunostaining obtained with two distinct antisera in neighboring sections (for KP: the Caraty#566 antiserum and the GQ2 antiserum; for NKB: the rabbit 681 and 682 antisera) [15]. A similar positive control experiment for SP was carried out by replicating the immunohistochemical staining using the rat and the rabbit SP primary antibodies.

Multiple positive and negative controls were used for immunofluorescent studies to rule out the possibility of artifactual colocalization results. The secondary antibodies were all raised in donkeys and recommended for multiple labeling by Jackson ImmunoResearch Laboratories. Negative control experiments included the omission of one of the primary antibodies from the procedure. Existence of many bright single-labeled, in addition to double- and triple-labeled structures also served as an endogenous control for the absence of antibody cross-reactions. Tyramide signal amplification was used sequentially twice, for the detection of SP and KP, respectively. To rule out the possibility that some of the KP signal was caused by residual peroxidase activity within SP-IR elements, control sections were processed in parallel with the only difference that the KP primary antiserum was left out from the procedure. These control sections remained entirely devoid of the KP signal (red Cy3 labeling). The colocalization phenomena were also confirmed in less complex dual-labeling experiments. In these cases, incubation in a mixture of primary antibodies was followed by a cocktail of FITC-conjugated (1:250) and Cy3-conjugated (1:1000) secondary antibodies (Jackson ImmunoResearch), according to the species used for raising the primary antibodies. Both the primary and the secondary antibody cocktails were used overnight at room temperature. In studies to colocalize KP with SP, dual-labeling with the rat SP antibodies and the sheep KP antiserum was reproduced with the combined use of the rabbit SP and the sheep KP antisera.

### Section mounting and coverslipping

Sections processed with peroxidase-based immunohistochemistry were mounted on microscope slides from Elvanol, air-dried, dehydrated with 95% (5 min), followed by 100% (2X5 min) ethanol, cleared with xylene (2X5 min) and coverslipped with DPX mounting medium (Sigma, St. Louis, USA). Immunofluorescent specimens were mounted from 0.1M Tris-HCl buffer (pH 7.6) and coverslipped with the aqueous mounting medium Mowiol.

### Analysis

Representative light microscopic images of immunoperoxidase labeled specimens were prepared with an AxioCam MRc 5 digital camera mounted on a Zeiss AxioImager M1 microscope using the AxioVision 4.6 software (Carl Zeiss, Göttingen, Germany). Confocal images were prepared with a Radiance 2100 (Bio-Rad Laboratories, Hemel Hempstead UK) confocal systems. Individual optical slices were collected for the analysis and for illustrations using the ‘lambda strobing’ function. This way, only one excitation laser and the corresponding emission detector are active during a line scan, to eliminate emission crosstalk between the fluorophores. Furthermore, the many heavily single-labeled neuronal structures in the confocal images served as intrinsic controls for the absence of bleed-through phenomena.

### Experimental design

#### Experiment 1. Distribution of SP immunoreactivity in the Inf

Triplets of sections were processed for the parallel detection and analysis of SP, KP and NKB immunoreactivities in the Inf. Peroxidase-based immunohistochemistry was used with the silver-gold-intensified nickel-diaminobenzidine chromogen.

#### Experiment 2. Quantitative analysis of SP-IR cell bodies in the Inf

Potential sex- and age-related differences in the incidence of SP-IR perikarya were addressed as in earlier studies that identified the sexual dimorphism [15,16] and aging-dependent changes in men [14] of KP-IR and NKB-IR cell bodies in the Inf. ‘Regional perikaryon density’ was defined by counting SP-IR perikarya (detected with the rabbit SP antiserum) in the Inf at 100X final magnification within a 0.25 mm^2^ counting area, with the aid of a 5X5 ocular grid. Each human subject was characterized with the highest number of immunoreactive cell bodies per counting area that was detectable in 1-3 sections [8,14-16]. The immunostained microscopic specimens were randomized, coded and analyzed by an investigator blind to the origin of samples. Comparison of groups (young men: N=8; aged men: N=6; postmenopausal women: N=8) was carried out with one-way ANOVA using the Statistica 8.0 software package (StatSoft, Inc, Tulsa, USA). Age effect was also addressed with regression analysis.

#### Experiment 3. Triple-immunofluorescent studies to detect SP within KP-IR and NKB-IR neuronal elements of the Inf

Triple-immunofluorescent studies followed by confocal microscopy were used to study and illustrate the colocalization between SP, KP and NKB immunoreactivities. The three fluorochromes were detected with the following laser lines and filters: 488nm for FITC, 543nm for Cy3 and 637nm for Cy5, with dichroic/emission filters 560nm/500–540nm for FITC, 650nm/560-610nm for Cy3 and a 660-nm-long pass filter for Cy5. The separately recorded green, red and far-red channels were merged and transferred into the red, green and blue channels of Adobe Photoshop (PSD) files, respectively. The extent of colocalization between the three neuropeptides was also quantified in the Inf of five postmenopausal women whose cell bodies showed the highest signal levels of all three immunoreactivities. Confocal images representing single optical slices were obtained using a 20X objective lense. Adobe Photoshop (PSD) files were analyzed by switching on and off between single channels and the colocalization percentages determined from a total of 777 labeled neurons. The results were expressed as the mean ±SEM of the five postmenopausal individuals and presented graphically in a pie diagram. Single-, double- and triple-labeled axons were identified and illustrated in single optical slices (<0.8 µm) prepared using a 60x oil immersion objective lense.

#### Experiment 4. Quadruple-immunofluorescent studies to investigate putative axo-axonal interactions between SP-IR fibers (some also containing KP and/or NKB) and GnRH-IR axons in the infundibular stalk

SP, KP and NKB immunoreactivities were detected with confocal microscopy as described for triple-immunofluorescent experiments, whereas the AMCA signal in GnRH neurons was visualized using a 405nm laser line with 500 nm/420-480 nm dichroic/emission filters. The SP, KP and NKB signals were collected into the red, green and blue channels, respectively, of Adobe Photoshop (PSD) files. The AMCA signal was transferred into the red and green channels of a separate layer to generate an orange pseudocolor. Axonal appositions were studied in single optical slices (<0.8 µm) obtained using a 60x oil immersion objective.

## Results

### Experiment 1. Distribution of SP immunoreactivity in the Inf

**Figure 1:**
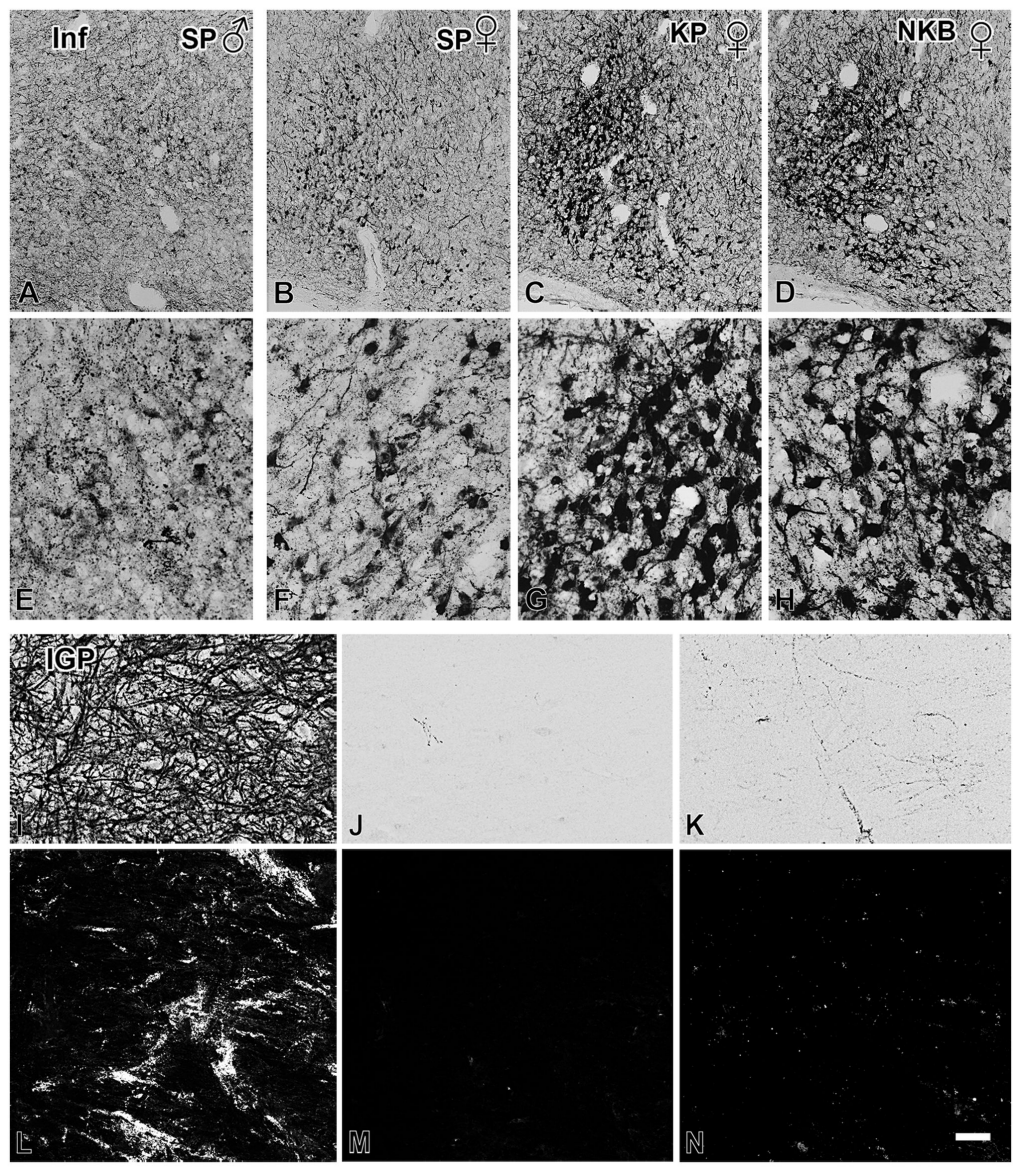
Overlapping distribution of Substance P, kisspeptin and neurokinin B immunoreactivities in the infundibular nucleus. Histological sections from a 69-year-old man (**A**, **E**) and a 57-year-old postmenopausal woman (**B**, **F**) illustrate higher numbers and heavier labeling of substance P (SP)-immunoreactive (IR) neurons in the infundibular nucleus (Inf) of women *vs*. men. The comparative mapping of SP (**B, F**; rabbit SP antiserum), kisspeptin (KP; **C, G**) and neurokinin B (NKB; **D, H**) immunoreactivities using the ABC method and silver-gold-intensified nickel-diaminobenzidine chromogen on tissue samples of the same female individual reveals a considerable overlap in the distribution of the three neuronal phenotypes. Note that other brain regions including the internal globus pallidus (IGP; **I-K**), exhibit unique and often non-overlapping immunoreactivity patterns for the same three neuropeptides. This site contains a dense plexus of single-labeled SP-IR axons (**I**; rabbit antiserum) and thus, can be used in triple-immunofluorescent experiments as an internal method control for antibody specificity. In triple-immunofluorescent experiments, the IGP exhibited heavy SP (**L**; rat SP antibodies) and little KP (**M**) and NKB (**N**) signals, as expected from the similar results of immuno-peroxidase experiments (**I**–**K**). Scale bar in **N** corresponds to 180 µm in **A-D**, 42 µm in **E-H**, 77µm in **I-K** and 50µm in **L-N**.

The topography of SP-IR neuronal cell bodies and fibers was in agreement with previous reports [26,27], with the majority of IR perikarya in the Inf (Figure 1A, B, E, F) and the infundibular stalk. The labeling intensity and number of somata were relatively low in men (Figure 1A, E) and high in histological samples obtained from postmenopausal women (Figure 1B, F). The distribution of labeled perikarya overlapped considerably with that of KP-IR (Figure 1C, G) and NKB-IR (Figure 1D, H) somata and the morphology of the three neuronal phenotypes was similar (Figure 1E-H).

Other brain sites included in the tissue sections exhibited unique labeling patterns for the three neuropeptides, indicating that the distributions of SP, KP and NKB do not overlap in all regions. Accordingly, the internal globus pallidus contained a very dense plexus of SP-IR axons (Figure 1I), as established previously [28], but only scattered KP-IR (Figure 1J) and relatively few NKB-IR (Figure 1K) fibers. This site was chosen for an important method control in subsequent triple-immunofluorescent experiments.

### Experiment 2. Quantitative analysis of SP-IR cell bodies in the Inf

**Figure 2:**
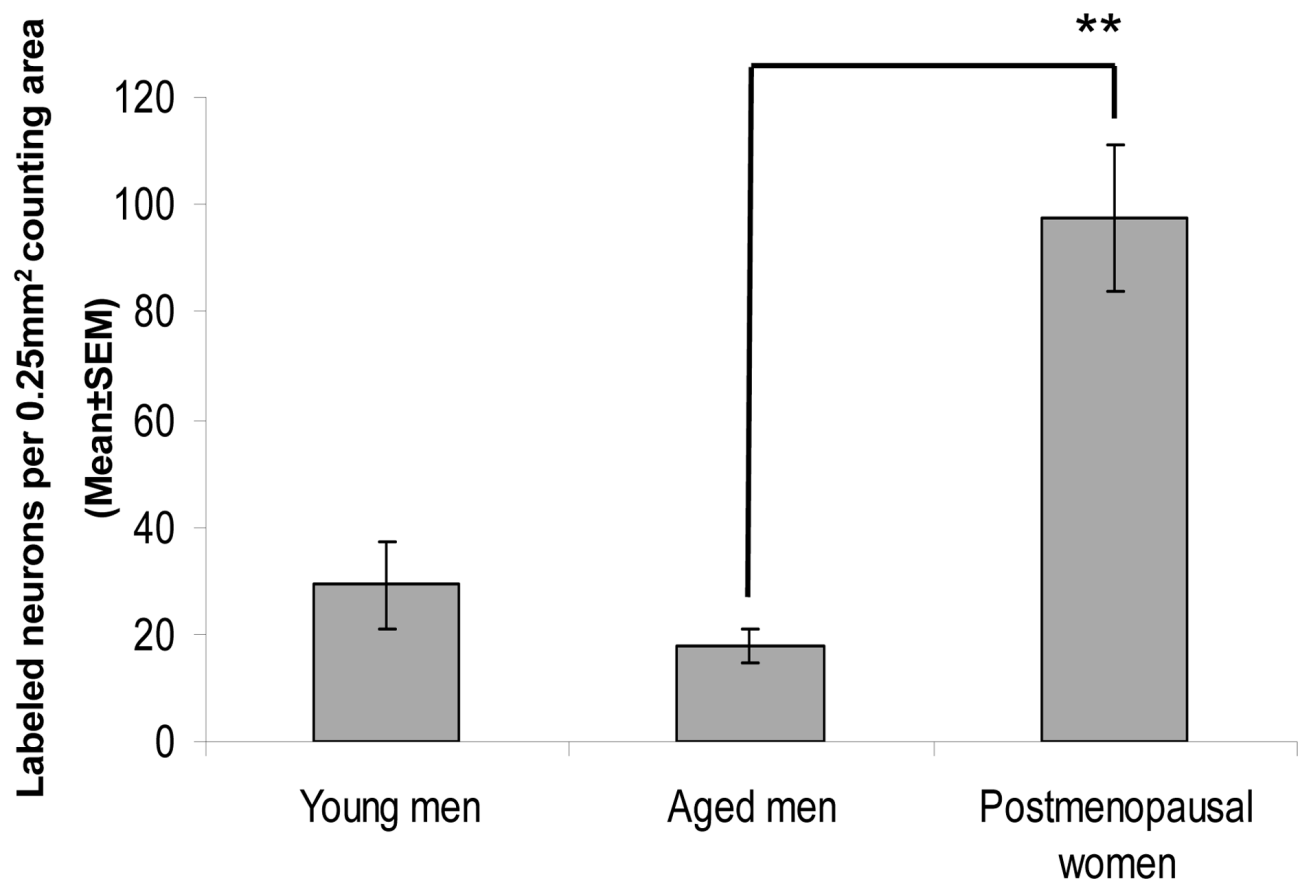
Regional abundances of Substance P-immunoreactive perikarya in the Inf of men below (‘young’ group) and above (‘aged’ group) 50 years of age and of postmenopausal women. The maximal number of immunoreactive cell bodies per 0.25mm^2^ counting area was determined with the aid of an ocular frame and used as the index of the regional neuron density. The abundance (regional density) of substance P-immunoreactive neurons defined this way is several-fold higher in postmenopausal women, compared with age-matched men, but does not differ significantly between men below (‘young’) and above (‘aged’) 50 years of age. *P<0.0005.

Quantitative analysis of SP-IR cell densities (maximal number of labeled somata per 0.25 mm^2^ counting area; Figure 2) identified a 5.4-times higher mean regional perikaryon density in the Inf of postmenopausal women, compared with men above 50 years of age. The sexual difference was highly significant (P=0.0004, by one-way ANOVA). Comparison of the two male age groups showed that although the mean regional density of labeled perikarya was slightly reduced from 29.1±8.2/0.25 mm^2^ in young (<50 ys) to 18.0±3.2/0.25 mm^2^ in aged (≥50 ys) men, the two groups did not differ statistically (P=0.29). Similarly, regression analysis showed no age effect on SP cell numbers in men (R^2^=0.1).

### Experiment 3. Localization of SP immunoreactivity within KP-IR and NKB-IR cell bodies and neuronal fibers

**Figure 3:**
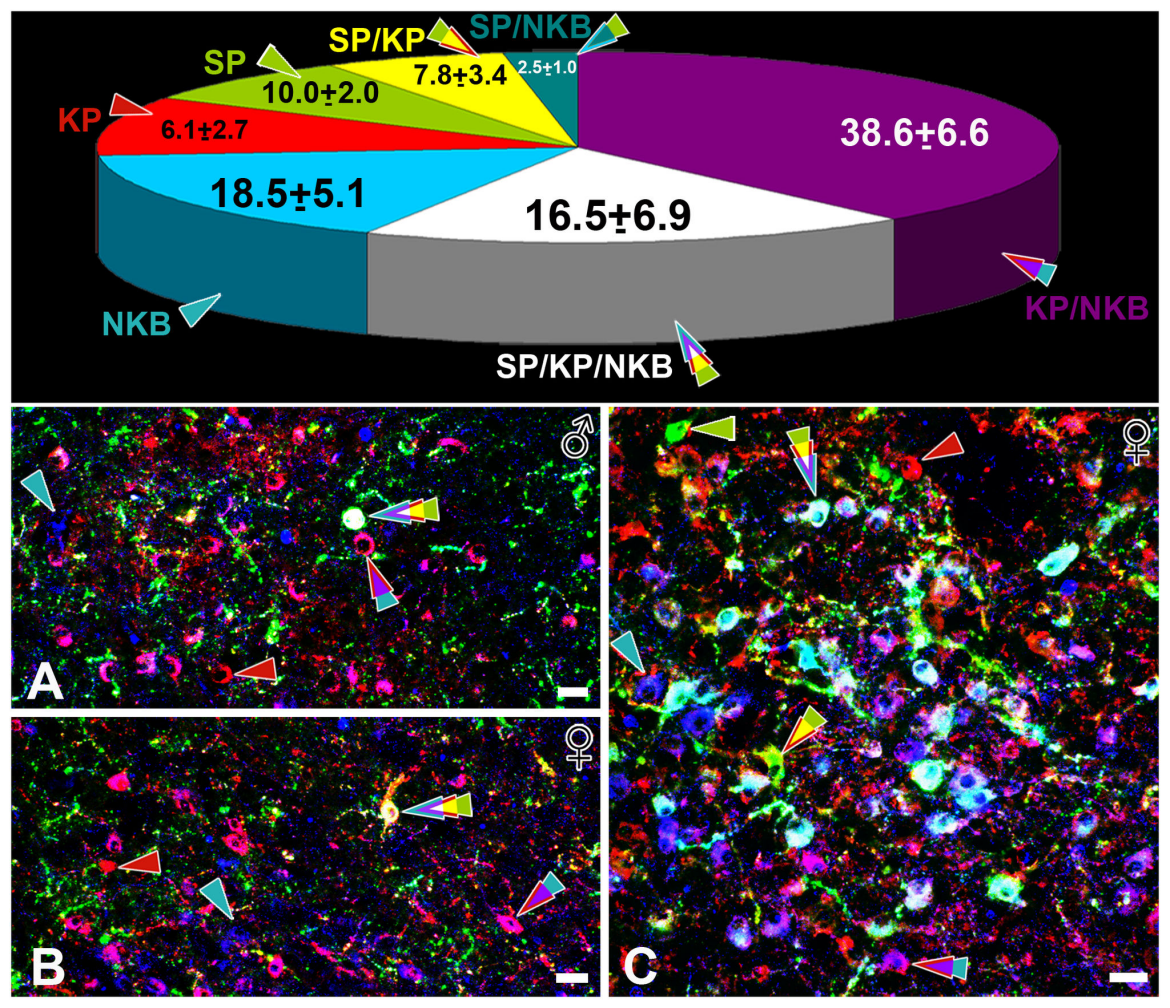
Triple-immunofluorescent localization of Substance P within kisspeptin-immunoreactive and neurokinin B-immunoreactive perikarya in the Inf. Triple-immunofluorescent studies of Substance P (SP; green), kisspeptin (KP; red) and neurokinin B (NKB; blue) immunoreactivities in the human infundibular nucleus reveal a substantial degree of colocalization between the three neuropeptides. Examples of single-, double- and triple-labeled perikarya are indicated by arrowheads. For color-coding, see pie chart at the top of the figure. Low-power images (**A**–**C**) illustrate that varying subsets of immunoreactive somata (three-color arrowheads; whitish neuronal labeling) contain all three neuropeptides in the Inf of men (**A**, 64 ys) as well as women (**B**, 58 ys; **C**, 57 ys). Note the presence of many single- and double-labeled cell bodies, in addition to these triple-labeled perikarya. The pie chart illustrates the mean incidences of the different cell phenotypes (mean±SEM of 5 postmenopausal women; 777 labeled neurons). 16.5±6.9% of the immunolabeled somata (representing 25.1±7.7% of the NKB-IR and 30.6±8.8% of the KP-IR perikarya) are triple-labeled (white color). Note that the most abundant labeled cell phenotype (38.6±6.6%) co-contains KP and NKB and is devoid of SP (purple color). Scale bars=25µm.

**Figure 4:**
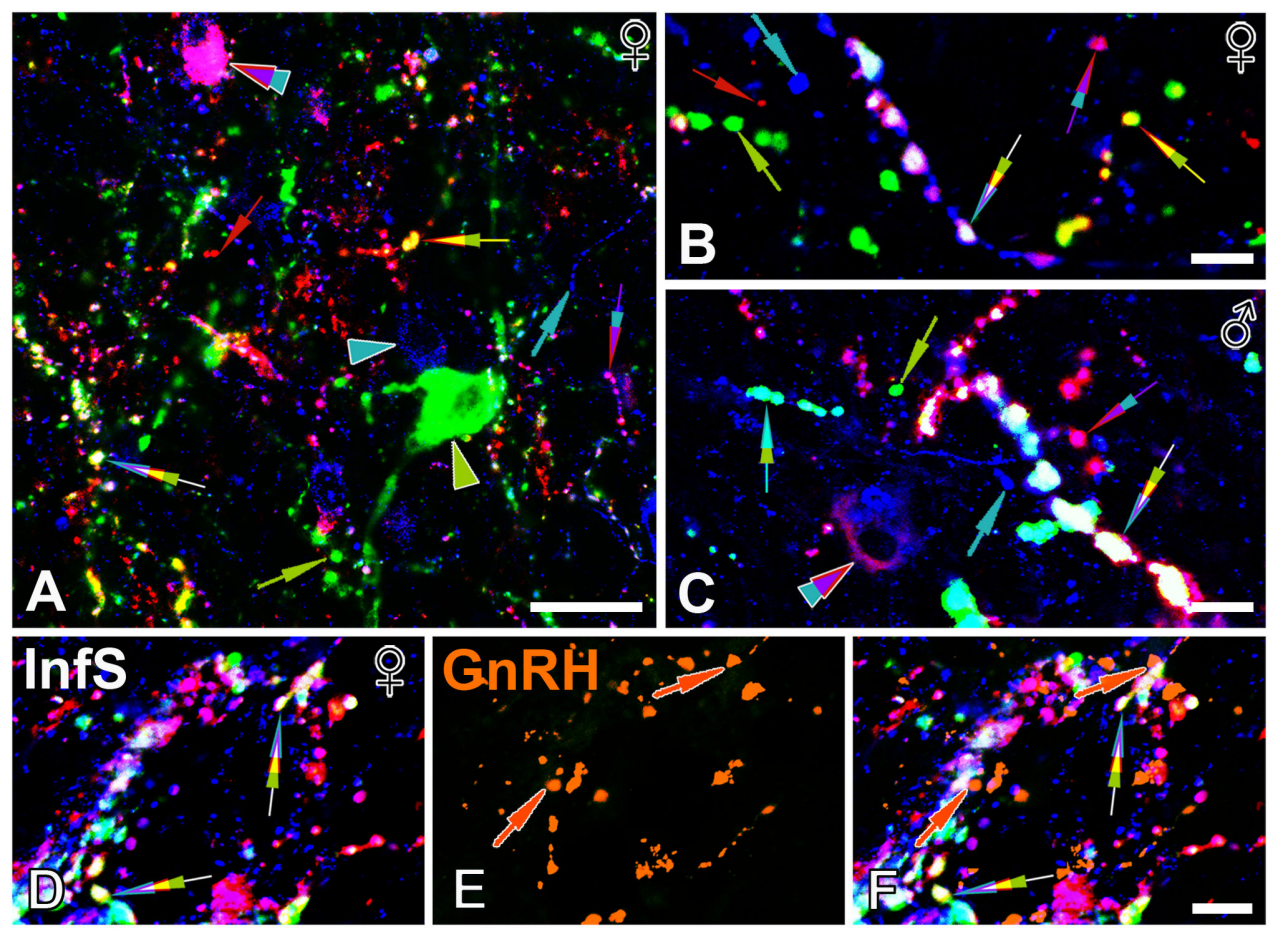
Triple-immunofluorescent localization of Substance P within kisspeptin-immunoreactive and neurokinin B-immunoreactive fibers in the Inf and the infundibular stalk. Representative confocal images (**A**, 83-year-old women; **B**, 69-year-old women; **C**, 64-year-old man) demonstrate that a subset of varicose peptidergic axons (indicated by the three-color arrows) in the Inf contain SP, KP and NKB, whereas the majority are single- or double-labeled (color-coded single and double arrows); arrowheads indicate labeled perikarya. The observation that colocalizations are only partial also provides an important endogenous method control for the triple-labeling technique. SP/KP fibers occur in yellow and SP/NKB fibers in turquoise color. High-power confocal images of quadruple-immunolabeled sections from the infundibular stalk (InfS; 83-year-old woman) in **D-F** illustrate a considerable degree of overlap between SP-IR, KP-IR and NKB-IR axon projections around portal capillary vessels. SP-IR axons often contain KP and NKB as well (**D**), suggesting the putative co-release of these neuropeptides into the hypophysial portal circulation. Orange pseudocolor in **E** labels GnRH-IR axons of the same field. Merged panel (**F**) reveals occasional appositions between SP-IR and GnRH-IR axons, which raise the possibility that SP influences GnRH neurosecretion at this site via axo-axonal interactions. In some of these appositions indicated by orange arrows the SP-IR axon is triple-labeled (SP/KP/NKB). For color-coding of the arrows and arrowheads, see pie chart at the top of Figure 3. Scale bars=25µm in **A** and 10µm in **B-F**.

The combined use of three distinct fluorochromes to visualize SP-IR, KP-IR and NKB-IR cell bodies and fibers in the infundibular region identified partial overlaps between the three different neuropeptide systems (Figures 3, 4). The use of tyramide signal amplification for SP and KP greatly enhanced the sensitivity of the immunofluorescent detection methods, which was critical in order to maximize the number of IR cell bodies. Confocal analysis of triple-labeled specimens identified single-, double-, and triple-immunolabeled somata (Figure 3A-C). In postmenopausal women where the sufficiently intense signals allowed the quantitative analysis of colocalization, SP immunoreactivity was detectable in 25.1±7.7% of NKB-IR and 30.6±8.8% of KP-IR perikarya. Out of all labeled neurons included in the analysis (777), KP/NKB double-IR somata exhibited the highest incidence (38.6±6.6%), followed by NKB-IR single-labeled (18.5±5.1%), KP/NKB/SP-IR triple-labeled (16.5±6.8%) and KP-IR single-labeled (10.0±2.0%) cell bodies. For detailed quantitative results, see pie diagram in Figure 3.

High-power confocal analysis of the Inf also demonstrated the presence of SP immunoreactivity in varying subsets of KP-IR, NKB-IR and KP/NKB-IR axon varicosities (Figure 4A-C). Such dual- and triple-labeled axons were evident in both females (Figure 4A, B) and males (Figure 4C).

Various positive and negative controls verified the specificity of the colocalization phenomena. The observation, that the coexpression of signals was only partial, provided an important intrinsic control for the specificity of labeling. In addition, the internal globus pallidus only contained a dense plexus of SP-IR single-labeled fibers (Figure 1L). This important internal control verified that the antibodies used to detect KP (Figure 1M) and NKB (Figure 1N) did not cross-react with the firstly detected SP-IR elements. Less complex dual-labeling approaches also revealed SP/KP-IR dual-labeled fibers and cell bodies using either the rat or the rabbit SP antibodies, in combination with the sheep KP antiserum. Similarly, dual-labeling control experiments also verified the presence of SP immunoreactivity in many NKB-IR neuronal elements with the combined use of the rat SP and the rabbit NKB antibodies.

### Experiment 4. Identification of SP-IR axons in the vicinity of GnRH terminals and hypophysial portal capillaries

Previously reported KP-IR and NKB-IR axon projections around the portal capillary system in the infundibular stalk [8,15] overlapped partly with the hypophysiotropic SP-IR axon projections [29] (Figure 4D). High-power images confirmed that some of the SP-IR axons were identical with KP-IR, NKB-IR and KP/NKB-IR axons, whereas SP-IR single-labeled axons were also encountered (Figure 4D). In quadruple-labeling experiments, the additional detection of GnRH-IR projections (Figure 4E) revealed GnRH-IR axons in the proximity of portal capillaries that were also surrounded by SP-IR axons (Figure 4F). Direct appositions between SP-IR and GnRH-IR axons (Figure 4F) raised the possibility that the infundibular stalk represents a putative site of axo-axonal interactions between SP and GnRH neurons, in addition to the previously reported axo-somatic and axo-dendritic contacts in other hypothalamic regions [27]. A subset of SP-IR axons in these appositions were also co-labeled for KP and NKB (Figure 4F).

## Discussion

In this study we provide immunohistochemical evidence that SP-IR neurons are present in higher numbers in the Inf of postmenopausal women compared with age-matched men. Our main observation is the frequent coexpression of SP immunoreactivity in KP-IR and NKB-IR neuronal cell bodies and fibers in the human infundibular region. Finally, we present morphological evidence for direct contacts between SP-IR fibers (some of which also contain KP and/or NKB) and the hypophysiotropic GnRH axon projections in the infundibular stalk.

### Functional significance of SP in human KP and NKB neurons

Depending on NK1 receptor sites that are currently unknown, SP may act pre- and/or postsynaptically to regulate synaptic transmission to GnRH neurons. The detection of SP in many KP-IR and NKB-IR fibers raises the possibility that some of the previously reported SP-IR contacts on GnRH neurons [27] could also contain KP and/or NKB. Provided SP has pre- or postsynaptic receptors in these connections, it may play a role as an important neuromodulator in this communication. Similarly, neurokinin A derived from the same gene may also occur in human KP and NKB neurons to act via the NK2 receptor. The presence of SP in considerable subsets of human KP and NKB neurons increases the complexity of the putative human pulse generator network. NKB signaling through NKB/NKB contacts [30] uses NK3 autoreceptors and this communication has been implicated in the mechanism of the pulsatile GnRH/LH secretion [3,4,31,32]. SP neurons exhibit a similar networking in humans [27], and our colocalization results suggest that NKB/NKB and SP/SP contacts may actually be partly identical. The important issue of whether or not, SP signaling through putative NK1 autoreceptors plays any role in the coordinated network activity of human KP, NKB and SP neurons, deserves further attention. Recent data from male mice by Navarro and colleagues indicate that intracerebroventricular administration of specific agonists to the NK1 and the NK2 receptors can similarly increase serum FSH and LH levels as the NK3 agonist senktide [33]. Furthermore, all three receptor agonists activated KP neurons of the ARC but not GnRH neurons of the preoptic area in electrophysiological experiments [33], raising the possibility that all three tachykinin peptide receptors serve as autoreceptors for KNDy neurons. The colocalization of SP with KP and NKB in the human infundibular stalk (present study) also suggests that these three neuropeptides are co-released to act on putative adenohypophysial receptors. Finally, the human infundibular stalk may be an additional site for complex axo-axonal interactions. SP may act here to modulate the secretory output of KP and NKB terminals via autocrine/paracrine mechanisms. Our observation that SP-IR axons (some being double- and triple-labeled for KP and NKB) were occasionally juxtaposed to GnRH-IR axons suggests that SP may modulate GnRH neurosecretion directly. To clarify the importance of these putative mechanisms, cellular and subcellular localization of the NK1 receptor will be crucial.

It will require clarification whether SP, we show here to occur in human KP and NKB neurons, is also present in NKB and KP (KNDy) neurons in different laboratory animals. The SP system of the ARC/Inf was shown to differ anatomically between humans and rodents in that SP neurons do not innervate the external zone of the median eminence in rat [29], but send abundant projections to the neurovascular zone in monkeys and humans [29]. The different contribution of SP to the function of rodent and human KP and NKB neurons also remains possible. With potential species differences in mind, it is interesting to note that SP signaling via the NK1 receptor does not seem to be absolutely necessary for reproduction in rodents, i.e. the Tac1-knockout mouse [34] which does not generate SP and neurokinin A and the NK1 receptor-knockout mouse [35] are both devoid of obvious reproductive deficiencies and remain fertile.

### The SP system in the human Inf is sexually dimorphic

In our present study we used a previously developed cell counting approach [8,14-16] to quantify and compare the regional abundance of SP-IR perikarya in the Inf of women and men. We found that the number of SP-IR somata was more than 5-times higher in postmenopausal women compared with age-matched men. The immunolabeled cell bodies of women also tended to stain much darker than in men. This sexual difference was highly reminiscent of similar observations we made earlier on the human KP and NKB neuronal systems [16]. An earlier *in situ* hybridization study [13] revealed higher preprotachykinin A and B mRNA levels in postmenopausal, compared with premenopausal, women. The postmenopausal changes are likely to result from the ovarian failure which reduces the negative regulation of the respective genes by estrogens. The issue of whether the sexual difference we observed in the regional density of SP-IR perikarya of aged individuals is entirely or only partly caused by the different sex steroid milieu of postmenopausal women and aged men, remains unresolved, given that samples from young female subjects were not available to allow similar sex comparisons. It will be conceptually important to clarify whether or not sexual differences already exist in young subjects as a result of the organizational effects of a differential sex steroid exposure of the two sexes during development. The estrogenic regulation of SP in various species, tissues and experimental settings seems to be complex. Negative regulation is indicated by the postmenopausal expansion of the human SP system in the Inf [13]. Furthermore, SP levels are also reduced in the rat anterior pituitary [36,37] or testicular Leydig cells [38] in response to estrogen treatment. Conflictingly, acute estrogen treatment upregulates SP in the guinea pig [39] and the rat [37,40,41] hypothalami. Evidence also exists to indicate that brain SP can be up- or down-regulated region-specifically [42]. Of note, *in situ* hybridization studies provided evidence for a robust negative estrogenic regulation of NKB expression in the rat ARC, but failed to reveal any estrogen- or estrous cycle dependence of the SP transcript [43], establishing a clear difference in the estrogen sensitivity of the two preprotachykinin genes in rats. Sex steroids also appear to regulate the two human transcripts differentially in the Inf. Accordingly, the NKB and SP transcripts were both higher in postmenopausal compared with premenopausal women, but the number of labeled neurons increased 15-fold in case of NKB and only 6-fold in case of SP [13].

### SP cell numbers do not show gross differences in the Inf between young and aged men

In our previous study we provided evidence that aging in men causes increased KP and NKB immunoreactivities in the Inf. Men above 50 years of age exhibited enhanced immunoreactive perikaryon densities, fiber densities and input incidences to GnRH neurons, compared with men below 50 years of age [14]. Furthermore, the incidence of KP expression in NKB neurons increased from 33-36% in young to 68% in aged men [14]. We have interpreted these aging-dependent changes as the consequences of reduced testosterone negative feedback in elderly men, in accordance with the known negative regulation of KP and NKB expression by testosterone in the mouse [31] and of KP expression in the monkey [44] ARC. It requires clarification to what extent testosterone regulates KP and NKB expression via acting directly on the androgen receptor or, after its aromatization to estrogen, on estrogen receptors. In our present study we addressed the possibility that similar aging-related changes also take place in the SP neuronal system. Unlike the number of KP-IR somata which largely increased with age and the number of NKB-IR somata which increased to a somewhat lower extent [14], we have found no evidence for any significant age-dependent change in the number of the detectable SP-IR cell bodies. In fact, the mean regional density of immunolabeled somata rather tended to slightly decrease with aging, although this change was not significant. One explanation for these negative data may be that SP expression is less sensitive to the declining testosterone feedback, compared with KP and NKB expression. Alternatively, it is also possible that estrogens and androgens regulate SP expression in the Inf differentially, and testosterone, unlike estrogens [13], enhances preprotachykinin A gene expression. This possibility gains support from earlier results showing that anterior pituitary SP levels are decreased by estrogen and increased by dihydrotestosterone treatment of adult rats [36]. In addition, SP immunoreactivity in Leydig cells was similarly decreased by estradiol treatment and restored after testosterone supplementation [38]. Finally, our approach of including in our counts the lightly-immunolabeled perikarya could not be sensitive enough to reveal mild aging-related alterations in the SP neuronal system. Of note, the low labeling intensity of SP-IR cell bodies in men prevented us from carrying out the reliable quantification of colocalization percentages in triple-immunofluorescent studies.

In summary, in this immunohistochemical study we provide evidence for the higher abundance of SP-IR cell bodies in the Inf of postmenopausal women compared with aged men. This sexual dimorphism may be partly caused by a reduced negative regulation of SP expression by estrogen in postmenopausal women. Furthermore, we demonstrate the colocalization of SP immunoreactivity in considerable subsets of KP-IR and NKB-IR neurons and their axon projections. The presence of SP in NKB and KP neurons increases the complexity of the putative pulse generator network in the human. Depending on the site/s of location of the SP receptor NK1, it is possible that SP modulates the effects of KP and NKB in axo-somatic and axo-dendritic afferents to GnRH neurons. Intrinsic SP may also regulate the activity and/or peptide release of the NKB and KP neuronal networks in an autocrine/paracrine manner. Furthermore, SP may also act in the infundibular stalk to influence the KP and NKB secretory output via additional autocrine/paracrine mechanisms or may regulate GnRH neurosecretion at this site directly from the GnRH terminals. A final possibility is the co-release of SP with KP and NKB into the hypophysial portal circulation to act on adenohypophysial gonadotrophs. Information about the cellular and subcellular localization of the SP receptor NK1 will be critical in order to assess the functional importance of these hypothetical mechanisms of action.
